# Cerebrovascular Function in the Large Arteries Is Maintained Following Moderate Intensity Exercise

**DOI:** 10.3389/fphys.2018.01657

**Published:** 2018-11-21

**Authors:** Jessica J. Steventon, Alex B. Hansen, Joseph R. Whittaker, Kevin W. Wildfong, Daniela Nowak-Flück, Michael M. Tymko, Kevin Murphy, Phil N. Ainslie

**Affiliations:** ^1^Neuroscience and Mental Health Research Institute, School of Medicine, Cardiff University, Cardiff, United Kingdom; ^2^Cardiff University Brain Research Imaging Centre, School of Physics and Astronomy, Cardiff University, Cardiff, United Kingdom; ^3^Centre for Heart Lung and Vascular Health, School of Health and Exercise Science, University of British Columbia Okanagan, Kelowna, BC, Canada

**Keywords:** P_ET_CO_2_, cerebrovascular reactivity, cerebral blood flow, exercise, haemodynamics, cerebral plasticity

## Abstract

Exercise has been shown to induce cerebrovascular adaptations. However, the underlying temporal dynamics are poorly understood, and regional variation in the vascular response to exercise has been observed in the large cerebral arteries. Here, we sought to measure the cerebrovascular effects of a single 20-min session of moderate-intensity exercise in the one hour period immediately following exercise cessation. We employed transcranial Doppler (TCD) ultrasonography to measure cerebral blood flow velocity (CBFV) in the middle cerebral artery (MCAv) and posterior cerebral artery (PCAv) before, during, and following exercise. Additionally, we simultaneously measured cerebral blood flow (CBF) in the internal carotid artery (ICA) and vertebral artery (VA) before and up to one hour following exercise cessation using Duplex ultrasound. A hypercapnia challenge was used before and after exercise to examine exercise-induced changes in cerebrovascular reactivity (CVR). We found that MCAv and PCAv were significantly elevated during exercise (*p* = 4.81 × 10^-5^ and 2.40 × 10^-4^, respectively). A general linear model revealed that these changes were largely explained by the partial pressure of end-tidal CO_2_ and not a direct vascular effect of exercise. After exercise cessation, there was no effect of exercise on CBFV or CVR in the intracranial or extracranial arteries (all *p* > 0.05). Taken together, these data confirm that CBF is rapidly and uniformly regulated following exercise cessation in healthy young males.

## Introduction

There is robust evidence that exercise is beneficial to vascular health. Randomized controlled trials indicate that exercise is as effective as drug interventions for reducing coronary heart disease mortality, treatment of heart failure and prevention of diabetes, and is more beneficial than drug treatment in stroke rehabilitation (Naci and Ioannidis, 2013). However, the underpinning mechanisms associated with this low-cost and accessible therapeutic intervention are not well understood.

Long-term exercise has been shown to induce angiogenesis, neurogenesis, and synaptic plasticity in the human and rodent brain (Isaacs et al., 1992; Cotman and Berchtold, 2002; van Praag et al., 2005; Black et al., 2008) which manifests as increased cerebral perfusion and metabolism (Nishijima and Soya, 2006; Kinni et al., 2011). Improving our understanding of how exercise promotes such cerebral vascular adaptations is crucial, given the increasingly recognized importance of vascular contributions to cognitive impairment and dementia (Gorelick et al., 2011) and the potential to optimize exercise to target the aging and diseased brain. The demand acute exercise places on the cerebrovasculature may be relevant to chronic adaptations as the acute demand may be a stimulus for adaptation and may reveal individual differences in the propensity for chronic adaptations.

The ability of the cerebral vasculature to adapt to changing metabolic demands depends on how readily it can alter its resistance, i.e., CVR. CBF is modulated by the PaCO_2_, which has a major influence on arteriolar diameter (Thomas et al., 1989; Ide and Secher, 2000; Ratanakorn et al., 2001). During exercise, both CBF and CVR to CO2 are increased (Rasmussen et al., 2006; Ainslie et al., 2008; Ogoh et al., 2008). Changes in CBF are intensity dependent, with CBFV in the MCA increasing linearly with exercise intensity until approximately 60–70% of maximal aerobic power, or VO_2_ max (Ide and Secher, 2000; Ogoh and Ainslie, 2009), before returning to baseline resting levels. Performing high-intensity exercise results in hyperventilation-induced hypocapnia, which subsequently causes cerebral vasoconstriction (Ogoh and Ainslie, 2009) and the observed return of CBFV to baseline values. Recent evidence suggests there is regional variation in the blood flow response during exercise (Sato et al., 2011; Smith et al., 2016, 2017; Washio et al., 2017). Similar to findings in the MCA, Sato et al. (2011) found that CBF in the ICA increased from rest to exercise at 60% of maximal aerobic power yet returned toward resting levels at 80% of maximal aerobic power. Crucially, the change in ICA CBF during graded exercise (40, 60, and 80% aerobic power) was correlated with the partial pressure of end-tidal CO_2_ (P_ET_CO_2_) (Sato et al., 2011). In contrast, in the external carotid, common carotid and VA, CBF increased proportionally with workload (Ainslie et al., 2008), suggesting a non-uniform effect of exercise across vascular territories.

Current understanding of cerebrovascular function in the period immediately following exercise cessation is limited, with previous studies showing equivocal results depending on the duration, intensity, and mode of exercise. For example, during the early recovery period directly after exercise, when blood pressure and HR are decreasing rapidly toward baseline levels, conflicting evidence exists for the stability of cerebral autoregulation (e.g., Koch et al., 2005; Ogoh et al., 2005, 2007; Murrell et al., 2007, 2009; Bailey et al., 2011) – the process of maintaining adequate and stable CBF during changes in blood pressure. Koch et al. (2005) observed alterations in cerebral autoregulation (decreased signal coherence and gain between blood pressure and CBFV) along with increased expired CO_2_ immediately following exhaustive resistance exercise. In contrast, cerebral autoregulation at the mean phase of the MCA velocity profile was found to be stable during the first 10 min following cessation of mild, moderate and heavy cycling (Ogoh et al., 2007). Beyond this early recovery phase, Willie et al. (2013) found CBFV was maintained in the MCA at 10-, 30-, and 60-min after aerobic exercise cessation, despite the presence of hypotension and exercise-induced hypocapnia. A lack of change in dynamic cerebral autoregulation change suggests that disturbances in vascular mechanisms are transient and recover within 10 min in the MCA, specifically. No studies to date have examined exercise recovery in cerebral arteries other than the MCA, despite evidence for regional variability during exercise (Sato et al., 2011).

In the recovery period following exercise, changes in CO_2_ have been shown up to 60-min following exercise cessation (Willie et al., 2013). Thus, it is necessary to understand how exercise-induced changes in arterial CO2 impact the cerebral vasculature during the post-exercise recovery period. There is evidence that CVR is affected by long-term exercise. Using TCD sonography, CVR was increased in the MCA following 12-weeks of exercise training in both young and older adults (Murrell et al., 2013), whilst in a separate TCD study of older adults, CVR was related to aerobic fitness in older adults (Barnes et al., 2013). In contrast and paradoxically, a blunted CVR response was observed in aged lifelong endurance runners compared to sedentary age-matched controls using magnetic resonance imaging. A proposed explanation for this blunting effect is the increased lifetime exposure to high levels of CO2 (i.e., exercise-induced hypercapnia) resulted in desensitization of the cerebrovasculature to CO2 stimulation. CVR has not been examined acutely during the post-exercise recovery phase.

In this study, we therefore sought to examine the immediate effects of a single session of moderate-intensity exercise on cerebrovascular function. To explore the hypothesis that regional variability in CBF would be observed post-exercise, we simultaneously measured CBFV in the MCA and PCA using transcranial Doppler ultrasonography (TCD) and blood flow in the ICA and vertebral arteries (VA) using Duplex ultrasound for up to 65 min after exercise cessation. To measure the dynamic vessel properties, CVR to hypercapnia was assessed based on the hypothesis that CVR would be increased during the post-exercise recovery period compared to baseline.

## Materials and Methods

**FIGURE 1:**
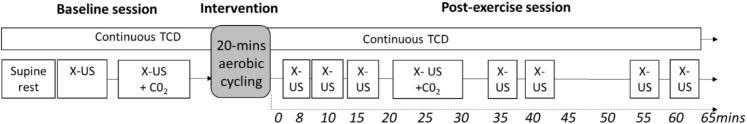
Study Design. Measures were recorded up to 65-min after exercise cessation. X-US, Doppler ultrasound to assess extracranial arteries; TCD, transcranial Doppler; X-US +CO_2_, Doppler ultrasound to assess cerebrovascular reactivity in the extracranial arteries with a hypercapnia challenge. Baseline ultrasound (X-US) was recorded for 5 continuous minutes after a minimum of 15-min supine rest. Cycling was performed on an upright ergometer.

The study design is detailed in Figure 1.

### Participants

Healthy male participants (*n* = 18; 26 ± 6.2 years old, range = 18–37 years) were recruited; demographic features are shown in Table 1. The inclusion criteria were: 18–55 years old, non-smoking, non-hypertensive and free from known neurological, respiratory and cardiovascular diseases. Participants were screened for contraindications to exercise and respiratory gas modulation. All participants were asked to refrain from vigorous physical activity, caffeine, and alcohol consumption for at least 12 h prior to experimentation. All subjects gave written informed consent in accordance with the Declaration of Helsinki. The protocol was prior approved by the clinical research ethics board at the University of British Columbia. Participants were instructed to lie supine for at least 15 min prior to beginning the study protocol.

**Table 1:** Subject demographics.

N	18
Age (years)	25.2 ± 1.1
BMI (kg/m^2^)	24.6 ± 0.6
SBP (mmHg)	115 ± 2
DBP (mmHg)	59 ± 1


*Values are mean ± SEM. BMI, body mass index; SBP: systolic blood pressure; DBP: diastolic blood pressure.*

### Exercise Intervention

Participants underwent 20-min of moderate-intensity aerobic cycling on an upright ergometer (Lode Ergometer, Lode, Groningen, Netherlands). To ensure a moderate-intensity aerobic intervention, a prescribed intensity of 50–70% of the maximal heart rate reserve (HRR) was determined using the Karvonen formula (American College of Sports Medicine et al., 2012) with maximum HR calculated as (220-age).

HRR=resting HR+(target HR% × [MaxHR−Resting HR])

Participants first rested on the ergometer for two-minutes before commencing a two-minute warm up at 25 watts. Following this, the resistance was adjusted to target the HRR, and was manually adjusted at two-minute intervals to ensure HR was maintained with the HRR zone. At two-minute intervals, whole blood capillary lactate concentration was collected from the left earlobe, blood pressure was measured and self-report ratings of perceived exertion were recorded using a 10-point Borg scale (Borg, 1974).

### Intracranial Blood Velocity

Cerebral blood flow velocity in the right MCA and left PCA (P1 segment) were measured using a 2-MHz pulsed TCD ultrasound system (PMD150B, Spencer Technologies, Redmond, WA, United States) using insonation techniques described in Willie et al. (2012), and identical to previous studies (Skow et al., 2013; Tymko et al., 2015). Mean CBFV was calculated from the envelope of the velocity tracings for both MCA and PCA.

### Extracranial Blood Velocity

Continuous diameter and blood flow recordings in the right ICA and left VA were obtained using a 10 MHz multifrequency linear array probe attached to a high-resolution ultrasound machine (Terason 3000, Teratech, Burlington, MA, United States) and using insonation techniques described in Thomas et al. (2015). Simultaneous measurements of blood vessel diameter and CBFV were recorded over a minimum of ten cardiac cycles with care taken to ensure probe position was stable. The sample volume was positioned in the center of the vessel and adjusted to cover the width of the vessel diameter.

All extracranial vascular images were stored for offline analysis using a custom-designed edge-detection and wall-tracking software described in depth elsewhere (Black et al., 2008; Willie et al., 2012). Mean blood flow was determined as half the time averaged maximum velocity multiplied by the cross-sectional lumen area, in line with Willie et al. (Evans, 1985; Willie et al., 2012; Thomas et al., 2015).

Extracranial blood velocity was not measured during exercise due to motion during upright cycling.

### Cardiorespiratory Measures

Cardiorespiratory measurements were sampled at 200 Hz using an analog-to-digital converter (Powerlab; AD Instruments; Colorado Springs, CO, United States) and analyzed offline (ADI LabChart version 7.1; AD Instruments, Colorado Springs, CO, United States). Participants breathed through a mouthpiece with nose clip, and respiratory gasses were recorded by a calibrated gas analyzer (ADI ML206; ADInstruments), with daily calibration to known gas concentrations performed prior to each test. P_ET_CO_2_ was calculated using peak analysis and was corrected for the daily atmospheric pressure.

Heart rate was measured by a three-lead electrocardiogram (ECG; ADI bioamp ML132; AD Instruments). Beat-to-beat mean arterial blood pressure (MAP), cardiac output and stroke volume were measured using finger photoplethysmography (Finometer Pro, Finapres Medical Systems, Amsterdam, NL), which was calibrated using the return-to-flow function prior to baseline data collection. Blood pressure accuracy was confirmed with manual sphygmomanometer.

### Steady State Iso-Oxic Elevations of P_ET_CO_2_

End-tidal gasses were controlled using a dynamic end-tidal forcing system previously described by Tymko et al. (2015). P_ET_O_2_, P_ET_CO_2_, inspiratory and expiratory tidal volume, breathing frequency and minute ventilation were determined for each breath in real time using custom software (Labview 13.0, National Instruments, Austin, TX, United States). To determine baseline end-tidal values, participants breathed room air until a steady-state was achieved (defined as ± 1 mmHg P_ET_CO_2_ and respiratory gasses being controlled and stable for at least three consecutive breaths).

### Hypercapnia Challenge

Dynamic end-tidal forcing was utilized to maintain P_ET_CO_2_ and P_ET_O_2_ at the baseline value on an individual basis for a minimum of one minute, during which, imaging of the extracranial arteries was conducted. Upon completion of the baseline stage, P_ET_O_2_ remained unchanged while P_ET_CO_2_ was elevated to +7 mmHg P_ET_CO_2_. After reaching steady-state P_ET_CO_2_, imaging of the extracranial arteries was conducted for two minutes. CBFV in the right MCA and left PCA were recorded continuously as described above.

### Statistical Analyses

Middle cerebral artery, PCAv and P_ET_CO_2_ were recorded continuously and statistically analyzed using a two-level approach in MATLAB R2012a (MathWorks, Natick, MA, United States). Time series were subjected to robust outlier detection, followed by temporal smoothing. A general linear model (GLM) was first fit to the individual subject data time series with the change in posture (supine to upright during transition to cycle ergometer) and P_ET_CO_2_ included in the model. Next, a one-sample *t*-test was performed on the regression coefficients, to assess the independent effect of (a) exercise on MCAv and PCAv, and (b) the effect of P_ET_CO_2_ on MCAv and PCAv.

For the extracranial arteries, 30-s averaging of the beat-by-beat data (velocity, diameter) was conducted and blood flow was calculated. Statistical analyses were performed on the data averaged across each recording period (Figure 1). Absolute values were assessed using a one-way repeated-measures analysis of variance (ANOVA; 8 levels: baseline, 8-, 15-, 20-, 40-, 45-, 60-, and 65-min post exercise) followed by pairwise *post hoc* comparisons, with a Bonferroni-adjustment for multiple comparisons. Relative change was assessed with a one-sample *t*-test and corrected for multiple comparisons using the false discovery rate at *p* < 0.05 (Benjamini and Hochberg, 1995). The assumption of sphericity was tested and where violated, the Greenhouse-Geisser adjusted values are reported.

Cerebrovascular reactivity was calculated in both absolute and relative terms (normalized to steady-state baseline) as the slopes of the linear regression of CBFV on P_ET_CO_2_ and % Δ CBFV on P_ET_CO_2_, respectively_,_ quantified on an individual basis. A one-way repeated-measures ANOVA was used to measure the effect of exercise. For relative measures of CVR, regional specificity was assessed for the intracranial and extracranial arteries separately (e.g., MCA and PCA as a within-subject variable, and ICA and VA as a within-subject variable) due to different acquisition methods.

## Results

### Exercise Intervention Checks

**FIGURE 2:**
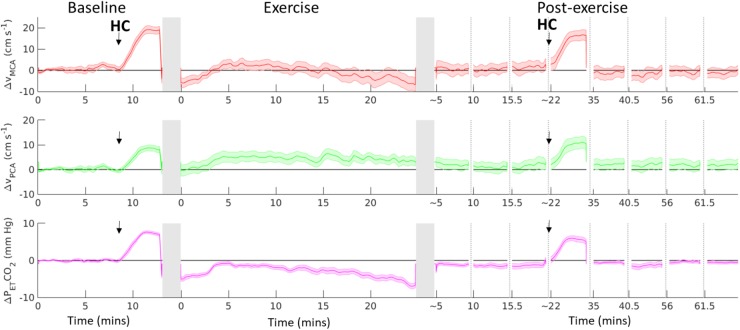
Change from baseline in MCA V_*mean*_ (red line), PCA V_*mean*_ (green line) and end-tidal CO_2_ (P_*ET*_CO_2_, purple line), before, during and after 20-min of moderate-intensity aerobic exercise. Black arrow indicates start of a hypercapnia (HC) challenge. Dotted vertical lines indicates discontinuity in the data, gray vertical columns indicate where the participant transferred from supine position to/from the cycle ergometer. Black horizontal line represents supine baseline. Data shown are mean ± S.E.M.

Results across the time series of the intervention are shown in Figure 2. Participants worked at an average 58.4 ± 1.2% of their maximal HR reserve. Average ratings of perceived exertion were 4 ± 0 in the legs, and 3 ± 0 for breathing, indicating “moderate” to “somewhat hard” exertion. Average blood lactate levels were 3.07 ± 0.57 mmoL (resting levels = 1.00 ± 0.1 mmoL). On average, participants cycled at a workload of 144 ± 9 watts, at a speed of 77 ± 2 revolutions per minute. MAP, HR and P_ET_CO_2_ were significantly elevated throughout the exercise intervention compared to the upright rest period on the ergometer (see Table 2 and Supplementary Figure S1).

**Table 2:** Cardiorespiratory and vascular response to acute exercise (experiment 2).

	Baseline	Exercise Intervention (duration; mins)					Post exercise (time; mins)						
			
		0	3	9	15	20	8	15	20	40	45	60	65
*Cardiorespiratory measures*													
HR, beats/min	60 ± 3	68 ± 5	102 ± 5^∗∗∗^	124 ± 5^∗∗∗^	141 ± 4^∗∗∗^	127 ± 4^∗∗∗^	75 ± 4^∗∗∗^	73 ± 4^∗∗∗^	71 ± 4^∗∗∗^	68 ± 4^∗∗^	66 ± 3^∗^	62 ± 3	69 ± 5
MAP, mmHg	88 ± 2	82 ± 2	112 ± 3^†††^	125 ± 2^†††^	126 ± 3^†††^	122 ± 3^†††^	83 ± 5	86 ± 5	90 ± 3	87 ± 3	87 ± 3	89 ± 3	86 ± 5
PETCO_2_, mmHg	42.9 ± 0.4	36.4 ± 0.7	40.5 ± 0.6^†††^	41.7 ± 0.7^†††^	40.7 ± 0.8^†††^	38.8 ± 0.8^†^	41.3 ± 0.6^∗^	41.1 ± 0.7	41.2 ± 0.8	41.8 ± 0.5	41.6 ± 0.6	42.0 ± 0.4	41.9 ± 0.5^∗^
Lactate, mmol/L		0.9 ± 0.1	1.8 ± 0.4	3.6 ± 1.0	3.9 ± 1.0	4.2 ± 0.8^†^						
*Vascular response in extracranial arteries*													
ICAv, cm/s^-1^	38.1 ± 2.1						40.3 ± 2.1	37.7 ± 1.9	37.8 ± 2.4	36.2 ± 2.0	37.3 ± 1.7	36.0 ± 1.9	35.4 ± 2.4
ICA flow, ml/min^-1^	261.5 ± 23.6						264.9 ± 27.0	235.9 ± 22.7	227.2 ± 22.8	222.3 ± 22.4	220.0 ± 17.2	222.2 ± 16.4	225.7 ± 24.1
ICA diameter, mm	5.49 ± 0.21						5.33 ± 0.22	5.27 ± 0.17	5.20 ± 0.22	5.14 ± 0.25	5.12 ± 0.14	5.18 ± 0.14	5.34 ± 0.20
VAv, cm/s-1	20.4 ± 1.5						19.5 ± 2.1	20.6 ± 2.0	20.7 ± 2.1	19.9 ± 2.0	19.3 ± 1.6	20.2 ± 1.9	19.2 ± 1.8
VA flow, ml/min^-1^	69.7 ± 79.8						75.1 ± 10.8	79.5 ± 10.9	77.2 ± 10.1	80.1 ± 10.9	82.8 ± 13.7	75.3 ± 9.7	73.9 ± 10.3
VA diameter, mm	3.77 ± 0.18						4.14 ± 0.23	3.71 ± 0.21	3.84 ± 0.17	3.94 ± 0.19	4.15 ± 0.21	4.02 ± 0.18	3.74 ± 0.29
*Vascular response in intracranial arteries*													
MCAv, cm/s^-1^	65.1 ± 0.7	59.3 ± 0.4	64.99 ± 0.6	66.7 ± 0.5	64.5 ± 0.6	61.5 ± 0.5	65.2 ± 0.5	64.9 ± 0.4	65.9 ± 0.4	63.3 ± 0.4	62.5 ± 0.5	63.8 ± 0.4	63.4 ± 0.3
PCAv, cm/s^-1^	39.9 ± 0.5	39.7 ± 0.3	43.3 ± 0.4	44.6 ± 0.4	44.3 ± 0.5	44.0 ± 0.5	41.7 ± 0.3	40.5 ± 0.2	41.2 ± 0.4	41.7 ± 0.3	41.4 ± 0.3	41.4 ± 0.3	41.5 ± 0.3


*Mean data ± SEMs are displayed at each time point. ^∗∗∗^*p* < 0.001. ^∗∗^*p* < 0.01 ^∗^*p* < 0.05 FDR-adjusted, between supine baseline and post-exercise time. ^†^refers to the statistical test between the upright rest period on the cycle ergometer (as opposed to supine rest), and time periods during the exercise intervention. ^†††^indicates *p* < 0.001.*

### Cardiorespiratory Response to Exercise

Table 2 shows the cardiorespiratory response during and after moderate-intensity exercise, averaged across each recording period. HR following exercise cessation was significantly elevated compared to supine baseline (*Greenhouse-Geisser corrected* F_2.6,45.0_= 10.37, *p* < 0.001, ε = 0.38), with *post hoc* tests showing HR remained significantly elevated for 45-min after exercise cessation (*p* < 0.05 Bonferroni-adjusted). Whereas MAP was significantly elevated during exercise, in the post exercise period (measured from 8-min post exercise cessation), MAP was not significantly changed compared to supine baseline (F_7,91_ = 0.44, *p* > 0.05). Compared to supine rest, P_ET_CO_2_ was significantly reduced during the post-exercise period (*Greenhouse-Geisser corrected* F_2.1,32.9_ = 4.80, *p* = 0.014, ε = 0.29), with *post hoc* tests showing participants were initially hypocapnic following exercise cessation (*p* = 0.012), and returned to resting levels of P_ET_CO_2_ 15-min after exercise cessation (*p* = 0.079).

### Cerebrovascular Response to Exercise

Figure 2 shows the absolute change in MCA_V_, PCA_V_, and P_ET_CO_2_ from baseline, including the hypercapnia challenge. On average across the 20-min exercise period, MCA_V_ and PCA_V_ were elevated by 17.2 ± 4.9% and 20.7 ± 4.4%, respectively, compared to the upright rest period. A *t*-test performed on the regression coefficients found that before accounting for P_ET_CO_2_, exercise had a significant effect on MCA_V_ (t_16_ = 5.50, *p* = 4.81 × 10^-5^, 95% CI [3.00 6.76]) and PCA_V_ (t_16_ = 4.70, *p* = 2.40 × 10^-4^, 95% CI [1.37 3.62]). After including P_ET_CO_2_ in the regression model and accounting for postural changes, P_ET_CO_2_ was found to have a significant effect on MCA_V_ (t_16_ = 14.94, *p* = 8.14 × 10^-11^, 95% CI = [1.66 2.21]) and PCA_V_ (t_16_ = 10.27, *p* = 1.89 × 10^-8^, 95% CI = [0.77 1.17]), whilst the effect of exercise on CBFV was no longer significant (MCA t_16_ = -0.49, *p* = 0.632, 95% CI [-2.51 1.57]; PCA t _16_ = -0.39, *p* = 0.702, 95% CI [-1.20 0.83]).

When comparing the post-exercise period to baseline, there was no effect of exercise on MCA_V_ before (t_16_ = 1.60, *p* = 0.13, 95% CI = [-7.38 1.04]), or after (t_16_ = -0.24, *p* = 0.82, 95% CI = [-4.95 3.88]) including P_ET_CO_2_ in the model. Similarly, there was no effect of exercise on PCA_V_ before (t_16_ = 0.44, *p* = 0.67, 95% CI = [-3.00 4.57]), or after (t_16_ = 1.21, *p* = 0.24, 95% CI = [-1.63 5.99]) including P_ET_CO_2_ in the model.

After accounting for postural change, P_ET_CO_2_ was found to account for 38.5 ± 16.9% of the variability in MCAv and 24.7 ± 16.9% of the variability in PCAv overall.

Absolute flow and CBFV values are shown in Table 2 for the extracranial feeding arteries (ICA, VA). There was a significant effect of time on absolute ICA velocity (F_7,105_ = 2.16, *p* = 0.043) with *post hoc* comparisons showing a significant difference between ICA velocity at 8 min post-exercise and 65-min post exercise (*p* = 0.048), however, this difference was not significant after accounting for the change in P_ET_CO_2_ (*p* = 0.069). Similarly, there was a main effect of time of absolute ICA blood flow (F_7,105_ = 2.67, *p* = 0.014); however, there were no pairwise differences after correcting for multiple comparisons (all *p* > 0.05). There was no effect of time on absolute VA velocity (*p* = 0.84) and no effect of time on relative ICA or VA flow (all *p* > 0.05) and on ICA and VA diameter (*p* > 0.05).

### Cerebrovascular Reactivity to Hypercapnia

During hypercapnia, both the absolute and relative rate of change in MCAv per mmHg P_ET_CO_2_ were not significantly different post-exercise (2.33 ± 0.17 cm/s, 3.65 ± 0.25% per mmHg, respectively) compared to baseline (2.44 ± 0.18 cm/s, 3.79 ± 0.27% per mmHg, respectively, all *p* > 0.05). Similarly, there was no statistical difference in CVR to CO_2_ for PCAv before (1.34 ± 0.12 cm/s, 3.41 ± 0.28% per mmHg) and after exercise (1.57 ± 0.16 cm/s, 3.92 ± 0.33% per mmHg, *p* > 0.05). The absolute and relative change in blood flow were also not significantly different pre- and post-exercise in the ICA (Baseline = 11.57 ± 3.64 ml/min, 4.60 ± 1.45% per mmHg; post-exercise = 7.51 ± 1.22 ml/min, 4.03 ± 0.88% per mmHg, *p* = 0.26 and 0.67, respectively) and in the VA (Baseline = 4.23 ± 0.89 ml/min, 5.47 ± 1.11%; post-exercise = 4.36 ± 1.59 ml/min, 5.33 ± 1.58%, *p* = 0.94 and 0.94, respectively) per mmHg P_ET_CO_2_.

In terms of regional differences in CO_2_ reactivity, there was no difference in relative CVR between the MCA and PCA (*p* = 0.81) and no interaction between vessel (MCA vs. PCA) and exercise effects (*p* = 0.23). Similarly, there was no difference in relative CVR for the ICA and VA (*p* = 0.88) and no interaction between vessel (ICA vs. VA) and exercise effects (*p* = 0.54).

## Discussion

This is the first known study to investigate cerebrovascular function in the middle and posterior cerebral arteries (MCA_V_ and PCA_V_, respectively) and in the extracranial cerebral arteries (ICA and VA) during the post-exercise recovery period. Based on evidence for regional variability in the vascular response during exercise (Sato et al., 2011), we hypothesized that regional variability in CBF would be observed post-exercise. We found that during exercise, significant changes in MCA_V_ and PCA_V_ were observed before changes in P_ET_CO_2_ were accounted for. However, after accounting for changes in P_ET_CO_2_ we instead found that CBFV in the MCA, PCA, ICA, and VA were stable compared to baseline, with changes in CBF being transient and returning to baseline rapidly and uniformly after exercise cessation in all measured vessels. Similar results have been reported previously in the MCA (Willie et al., 2013). Critically, P_ET_CO_2_ was measured and included in the statistical analyses as a regressor in this study, which allowed us to show for the first time that observed changes in CBFV during exercise were completely accounted for by exercise-induced changes in P_ET_CO_2_; despite an initial period of hypocapnia post exercise, CBF and CBFV remained stable compared to baseline.

We directly manipulated CO_2_ levels through the use of a hypercapnic challenge to measure CVR, which has not previously been examined acutely during the post-exercise recovery phase. We hypothesized that CVR to hypercapnia would be increased post-exercise compared to baseline, based on evidence of increased CVR during exercise (Ogoh et al., 2008) and following exercise training (Murrell et al., 2013). In contrast to our hypothesis, we found that CVR in the large cerebral vessels was stable during the post-exercise recovery period. The lack of a post-exercise effect was observed under normotensive conditions after 20-min of moderate intensity aerobic cycling. The duration of the exercise session was selected to be a feasible time period for future therapeutic studies with clinical and aged groups; however, this duration and intensity may account for the results observed. Previous work using TCD to measure the effect of exercise-induced syncope after four hours of prolonged exercise reported changes in MCA velocity and CVR (Murrell et al., 2013), whereas 40-min of similar-intensity exercise was also found to produce no alterations in cerebral autoregulation in the MCA at 10, 30-, and 60-min after exercise cessation under pharmacologically-induced hypotensive conditions (10 mmHg in blood pressure) (Willie et al., 2013). Taken together, this suggests that the duration of exercise influences the post-exercise recovery response. For shorter exercise durations and in the absence of syncope, disturbances in cerebral autoregulation (Koch et al., 2005) are transient and recover by post 10 min in large arteries.

The results of the current study are notably restricted to a cohort of healthy normotensive adults aged under 40 years old, whilst the effect of age on exercise-related cerebrovascular function remains equivocal to date. During exercise, age effects in mean CBF velocity in the MCA have been reported (Heckmann et al., 2003; Marsden et al., 2012; Fisher et al., 2013; Flück et al., 2014) along with enhanced cerebral vasoconstriction at moderate-to-high exercise workloads specifically in older groups (Ogoh et al., 2011; Fisher et al., 2013). In contrast, other studies have suggested a more marked response in young cohorts, for example, during maximal exercise, older adults were found to have a blunted hyperventilation response resulting in less hypocapnia-induced cerebral vasoconstriction (Marsden et al., 2012), whilst cerebral autoregulatory capacity was found to be retained but delayed in response to ergometer stress in healthy normotensive elderly subjects compared with healthy young subjects (Heckmann et al., 2003). In contrast, the chronic, or long-term effects of exercise appear to be less affected by age, with a training-induced elevation in CVR observed in both young and older cohorts (Murrell et al., 2013), along with a consistent elevation in MCAv in in endurance-trained men compared to sedentary men irrespective of age (Ainslie et al., 2008). Methodologically, the validity of the equation commonly used to estimate maximal HR and as a basis for prescribing exercise programs has been shown to underestimate maximal HR in older adults (Tanaka et al., 2001), which may contribute to the difficulty in making comparisons across the lifespan and is an important consideration when evaluating observed age effects.

### Methodological Considerations

A strength of this study is the combination of Duplex ultrasound with TCD sonography to examine both CBF velocity and diameter in the large cerebral arteries with real-time resolution. Most previous exercise studies have solely relied on TCD methods (Murrell et al., 2013; Willie et al., 2013), however, TCD only gives a reliable index of CBF if the diameter of the insonated artery is constant. Exercise alters PaCO_2_ and increases arterial pressure by sympathetic activation, and previous work has shown that CBF velocity derived from TCD underestimates CBF during modest hypercapnia and hypocapnia (Coverdale et al., 2014). Moreover, a recent study employing magnetic resonance imaging at high spatial resolution showed a 2% decrease in MCA cross-sectional area during rhythmic handgrip exercise (Verbree et al., 2017), suggesting that diameter constancy cannot be assumed during exercise, and thus represents a confound for TCD exercise studies. Here, we measured diameter in the ICA and VA simultaneously at baseline and during the post-exercise recovery period, and found that along with CBF velocity, diameter did not change during the post-exercise recovery period.

In order to reduce between-subject variability in autonomic regulation (Rossy and Thayer, 1998; Kuo et al., 1999; Lutfi and Sukkar, 2011), only male participants were recruited in the current study. Whereas there is no evidence to date to suggest sex differences occur in the cerebrovascular response to exercise, sex differences have been shown in resting cerebral haemodynamics (Tarumi et al., 2014) and in cerebrovascular CO_2_ reactivity (Kastrup et al., 1998), and studies with the statistical power required to examine sex differences are warranted.

A methodological consideration in this study is the prescription of moderate-intensity exercise with a range of 50–70% of the maximal HRR which may have introduced unnecessary inter-subject variability in the results. An additional consideration is the use of end-tidal CO_2_ (P_ET_CO_2_) as a statistical regressor across the time course. P_ET_CO_2_ was used as a non-invasive estimate of PaCO_2_ to avoid the invasive procedure of sampling arterial blood. However, whereas P_ET_CO_2_ is a good index of PaCO_2_ during resting conditions, P_ET_CO_2_ becomes higher than PaCO_2_ when metabolic CO_2_ production and ventilation are increased during exercise and therefore PaCO_2_ may be overestimated during the exercising period (Dubois et al., 1952; Jones et al., 1966, 1979). Given that the primary focus of this study was on changes occurring during the post-exercise recovery period rather than during exercise, and that results obtained during exercise replicate previous findings (Ogoh and Ainslie, 2009), this methodological issue is not likely to affect the interpretation of the results.

## Conclusion

Cardiorespiratory recovery following moderate-intensity exercise does not map onto cerebrovascular recovery. For the first time, we show that CBF in the large cerebral arteries recovers rapidly and uniformly after cessation from moderate intensity exercise and remains stable during the post-exercise recovery period. Similarly, we found no change in CVR approximately 25-min after exercise cessation. Future work is required to ascertain if cerebrovascular recovery is similarly efficient in aged populations, which is of particular relevance given the importance of vascular contributions to dementia and the potential for exercise as a therapeutic for cognitive aging.

## Data Availability Statement

The raw data supporting the conclusions of this manuscript will be made available by the authors, without undue reservation, to any qualified researcher.

## Author Contributions

JS, AH, DN-F, MT, KM, and PA contributed toward the research project organization and execution, and reviewed the manuscript. JS, KM, and PA additionally contributed toward the research project conception, statistical analysis design and review. JW contributed toward the statistical analysis design, execution and along with manuscript review. KW contributed toward the research execution.

## Conflict of Interest Statement

The authors declare that the research was conducted in the absence of any commercial or financial relationships that could be construed as a potential conflict of interest.

## Abbreviations

CBF: cerebral blood flow
CBFV: cerebral blood flow velocity
CVR: cerebrovascular reactivity
HR: heart rate
ICA: internal carotid artery
MAP: mean arterial pressure
MCA: middle cerebral artery
PaCO_2_: Partial pressure of arterial carbon dioxide
PCA: posterior cerebral artery
PCO_2_: partial pressure of carbon dioxide
P_Et_CO_2_: partial pressure of end-tidal respiratory carbon dioxide
TCD: transcranial doppler
VA: vertebral artery
