# Supplementary figures and images for: Cerebrovascular Function in the Large Arteries Is Maintained Following Moderate Intensity Exercise
Source: Front Physiol. 2018 Nov 21;9:1657. doi: 10.3389/fphys.2018.01657 (PMC6258791; doi:10.3389/fphys.2018.01657)

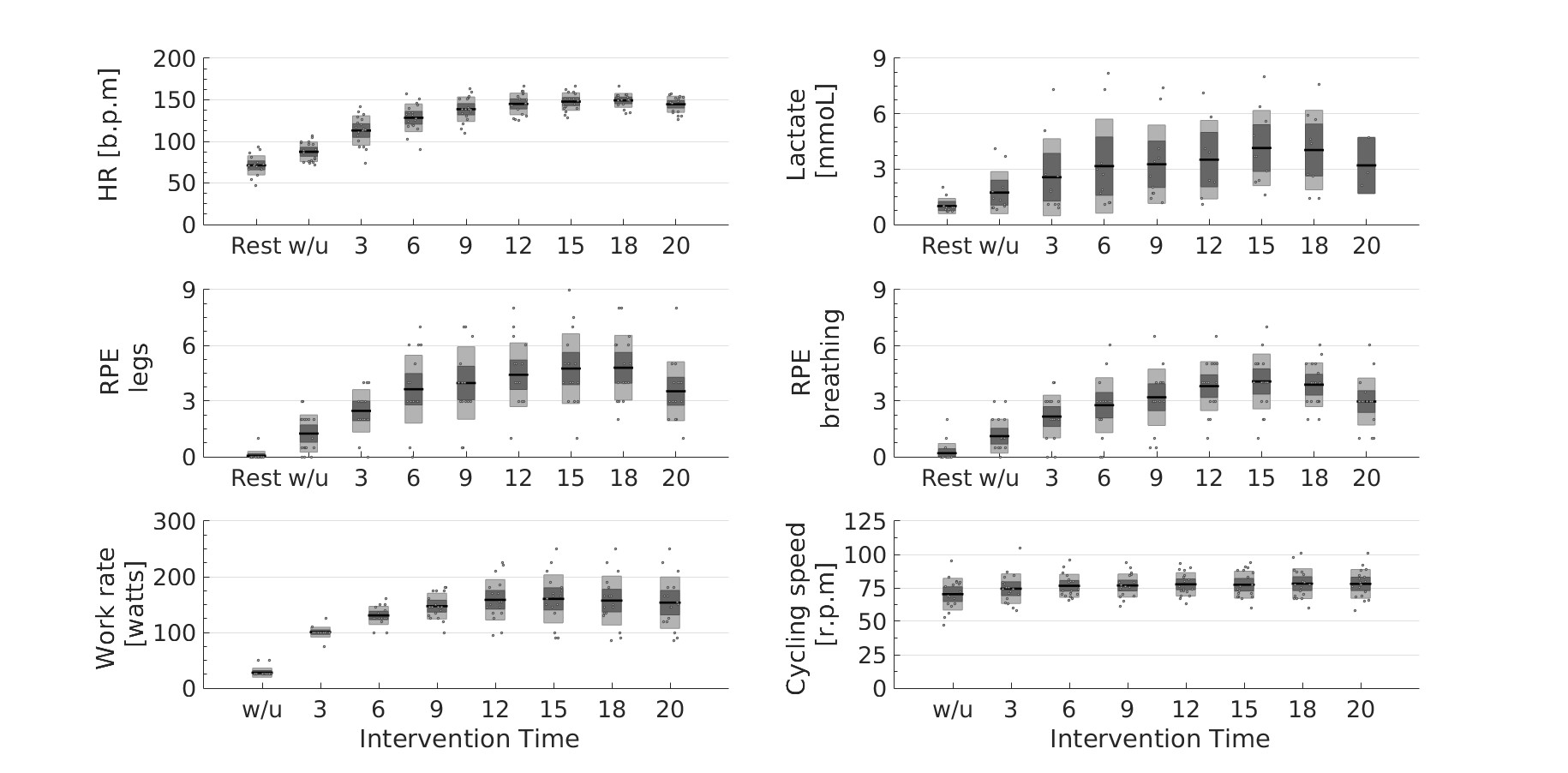

Supplement: Supplementary file 1 [file Image_1.JPEG]
