# Supplementary material for: Cerebrovascular Function in the Large Arteries Is Maintained Following Moderate Intensity Exercise
Source: Front Physiol. 2018 Nov 21;9:1657. doi: 10.3389/fphys.2018.01657 (PMC6258791; doi:10.3389/fphys.2018.01657)
Supplement: Supplementary file 2 [file Table_1.DOCX]

Supplementary Figure 1 Cardiac, metabolic and perceptual responses recorded during the exercise intervention delivered in experiment 1. The x-axis depicts the duration of the intervention (minutes), including a warm-up (w/u) period. Black solid line represents median with 95% CI (grey region) HR: heart rate, RPE: rating of perceived exertion.
